# Vaccine hesitancy among hospital workers

**DOI:** 10.1192/j.eurpsy.2022.1370

**Published:** 2022-09-01

**Authors:** A.H.I. Abu Shehab, C. Cruceanu, A.B. Ciubara, A.V. Gurita, L. Luca, A. Ciubara

**Affiliations:** 1 “Elisabeta Doamna” Psychiatry Hospital of Galati, Psychiatry Department, Galati, Romania; 2 Elisabeta Doamna Psychiatry Hospital of Galati, Psychiatry Department, Galati, Romania; 3 ’Dunerea de jos’’ University of Galati, Orthopedics, Galati, Romania; 4 ’Elisabeta Doamna’’ Psychiatry Hospital of Galati, Psychiatry Department, Galati, Romania; 5 University of Medicine and Pharmacy “Grigore T. Popa”, Psychology, Iasi, Romania; 6 ’Dunerea de jos’’ University of Galati, Psychiatry Department, Galati, Romania

**Keywords:** vaccine hesitancy, SARS-CoV-2, pandemic, Covid-19

## Abstract

**Introduction:**

Vaccine hesitancy is a serious issue and it affects the scientific achievements of health. This phenomenon has begun to be studied more often in health care workers, to find its determining factors.

**Objectives:**

The aim was to determine the percentage of hospital workers who got vaccinated against the infection with SARS-CoV-2.

**Methods:**

Beginning with October 2021, we conducted an online questionnaire in which 57 hospital workers participated. Preliminary results allowed us to assess the rate of vaccine hesitancy among this group.

**Results:**

Out of the 57 hospital workers, the majority were vaccinated (n=45, 78.94%) in comparison to less than a quarter (n=12, 21.05%) that refused vaccination. The group of hospital workers included mostly nurses ( n=21, 36.84%). Also, 12 psychologists (21.05%), 11 doctors (19.29%), and 10 students (17.54%) were included. Among the cases that did not accept getting vaccinated against COVID-19, the highest percentage was occupied by nurses (n=9, 15.78%). Moreover, there were only one doctor and one psychologist who did not get vaccinated.

**Conclusions:**

In the current pandemic times, the hesitancy and refusal of vaccination prove to be very challenging. It is important to explore their reasons and to promote health education programs.

**Disclosure:**

No significant relationships.

